# Developing Clinical Artificial Intelligence for Obstetric Ultrasound to Improve Access in Underserved Regions: Protocol for a Computer-Assisted Low-Cost Point-of-Care UltraSound (CALOPUS) Study

**DOI:** 10.2196/37374

**Published:** 2022-09-01

**Authors:** Alice Self, Qingchao Chen, Bapu Koundinya Desiraju, Sumeet Dhariwal, Alexander D Gleed, Divyanshu Mishra, Ramachandran Thiruvengadam, Varun Chandramohan, Rachel Craik, Elizabeth Wilden, Ashok Khurana, The CALOPUS Study Group, Shinjini Bhatnagar, Aris T Papageorghiou, J Alison Noble

**Affiliations:** 1 Nuffield Department of Women's and Reproductive Health, University of Oxford Oxford United Kingdom; 2 Institute of Biomedical Engineering, University of Oxford Oxford United Kingdom; 3 Translational Health Science and Technology Institute Faridabad India; 4 The Ultrasound Lab New Delhi India; 5 See Acknowledgments; 6 Oxford Maternal & Perinatal Health Institute, Green Templeton College, University of Oxford Oxford United Kingdom

**Keywords:** ultrasound, obstetrics, artificial intelligence, machine learning, data annotation

## Abstract

**Background:**

The World Health Organization recommends a package of pregnancy care that includes obstetric ultrasound scans. There are significant barriers to universal access to antenatal ultrasound, particularly because of the cost and need for maintenance of ultrasound equipment and a lack of trained personnel. As low-cost, handheld ultrasound devices have become widely available, the current roadblock is the global shortage of health care providers trained in obstetric scanning.

**Objective:**

The aim of this study is to improve pregnancy and risk assessment for women in underserved regions. Therefore, we are undertaking the Computer-Assisted Low-Cost Point-of-Care UltraSound (CALOPUS) project, bringing together experts in machine learning and clinical obstetric ultrasound.

**Methods:**

In this prospective study conducted in two clinical centers (United Kingdom and India), participating pregnant women were scanned and full-length ultrasounds were performed. Each woman underwent 2 consecutive ultrasound scans. The first was a series of simple, standardized ultrasound sweeps (the CALOPUS protocol), immediately followed by a routine, full clinical ultrasound examination that served as the comparator. We describe the development of a simple-to-use clinical protocol designed for nonexpert users to assess fetal viability, detect the presence of multiple pregnancies, evaluate placental location, assess amniotic fluid volume, determine fetal presentation, and perform basic fetal biometry. The CALOPUS protocol was designed using the smallest number of steps to minimize redundant information, while maximizing diagnostic information. Here, we describe how ultrasound videos and annotations are captured for machine learning.

**Results:**

Over 5571 scans have been acquired, from which 1,541,751 label annotations have been performed. An adapted protocol, including a low pelvic brim sweep and a well-filled maternal bladder, improved visualization of the cervix from 28% to 91% and classification of placental location from 82% to 94%. Excellent levels of intra- and interannotator agreement are achievable following training and standardization.

**Conclusions:**

The CALOPUS study is a unique study that uses obstetric ultrasound videos and annotations from pregnancies dated from 11 weeks and followed up until birth using novel ultrasound and annotation protocols. The data from this study are being used to develop and test several different machine learning algorithms to address key clinical diagnostic questions pertaining to obstetric risk management. We also highlight some of the challenges and potential solutions to interdisciplinary multinational imaging collaboration.

**International Registered Report Identifier (IRRID):**

RR1-10.2196/37374

## Introduction

### Background

Every year between 250,000 and 300,000 women die during pregnancy or following childbirth, and approximately 2.5 million neonates die within the first 28 days of life [1]. Most of these deaths occur in low-resource settings and can be prevented by timely access to evidence-based interventions. To help mitigate these deaths and the associated large burden of morbidity, the World Health Organization recommends a package of antenatal care [2] that includes an ultrasound before 24 weeks of gestation. The recommendations for the antenatal ultrasound are to assess fetal cardiac activity, fetal number and chorionicity, gestational age and fetal size, placental appearance and location, and a basic anomaly screen [2]. In addition to early screening, routine assessment of fetal malpresentation near term is also effective in reducing morbidity and mortality [3].

Significant barriers remain to the universal access to antenatal ultrasound. Primarily, these are related to procuring and maintaining ultrasound equipment and a lack of trained personnel [4]. The first of these barriers is being addressed through technological advances in low-cost, handheld ultrasound devices, making point-of-care ultrasound more accessible. However, implementation remains limited due to insufficient numbers of trained health care providers. In addition, training in obstetric ultrasound is lengthy, costly, and difficult to scale up. Several research efforts have described how teleradiology or automated solutions might overcome these obstacles through the use of simple obstetric ultrasound protocols using portable devices [5-10].

### Objectives

To contribute to the improvements in antenatal care and pregnancy risk assessment for women in low-resource settings, we are undertaking the Computer-Assisted Low-Cost Point-of-Care UltraSound (CALOPUS) project. This brings together experts in machine learning and clinical obstetric ultrasound in an international collaboration (Multimedia Appendix 1). The aim of this study is to develop and evaluate simple-to-use clinical protocols and machine learning–based decision-making tools for nonexpert users, which are designed to be functionally suitable for implementation at scale in underserved regions.

In this paper, we describe our approach to working toward automating the requirements of a basic ultrasound examination [2,11]. Well-designed acquisition and curation of data and their careful annotation are crucial in such studies. The aims of this paper are to report the development of optimal clinical acquisition and image annotation protocols suitable for automated analysis and to describe our experience and share learning regarding ultrasound video acquisition and annotation in a multisite setting.

## Methods

### Study Overview

The ultimate objective of the CALOPUS study is to develop machine learning models based on simplified obstetric ultrasound to predict pregnancy risk factors, such as the detection of noncephalic presentation or a low-lying placenta. The primary objectives of this phase of CALOPUS are as follows:

1. To develop an optimal clinical acquisition protocol suitable for automated analysis that can be obtained by a minimally trained health care provider
2. To capture ultrasound videos and perform video annotation to develop a data set suitable for machine learning
3. To advance capabilities in real-time ultrasound video partitioning to prevent fetal sex determination, which is an important prerequisite for the global dissemination of ultrasound

This prospective study is an interdisciplinary international collaboration among the Translational Health Science and Technology Institute, Delhi, India; the Civil Hospital, Gurugram, Haryana, India; the Institute of Biomedical Engineering and the Nuffield Department of Women’s and Reproductive Health, University of Oxford, United Kingdom; and the Women’s Centre, John Radcliffe Hospital, Oxford, United Kingdom.

### Setting, Study Design, and Participants

**Figure 1:**
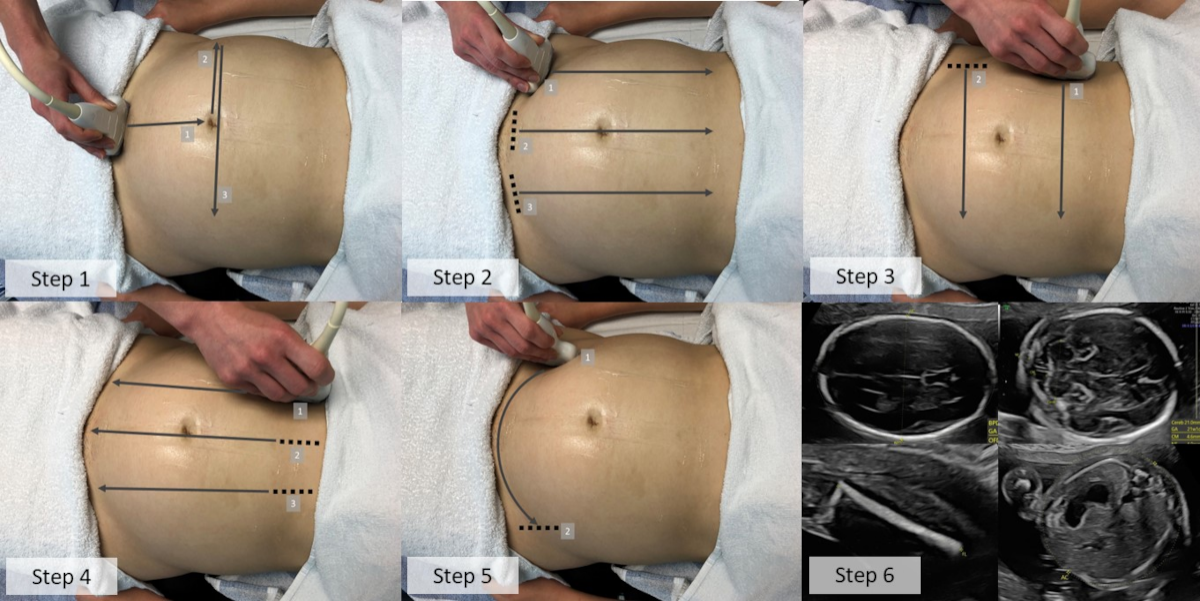
The Computer-Assisted Low-Cost Point-of-Care UltraSound protocol, developed on the basis of the studies described (see text).

In the 2 hospitals, the participating pregnant women were scanned in a room set up with an ultrasound machine configured to record full-length scans by screen capture. Each woman underwent 2 consecutive ultrasound scans. The first was our CALOPUS protocol, which consists of a series of simple, standardized ultrasound sweeps (Figure 1), immediately followed by a routine, full clinical ultrasound examination that served as the comparator. All scans were performed by trained sonologists (both sonographers and medical doctors trained in obstetric ultrasound) on identical GE Voluson E8 (General Electric Healthcare) ultrasound machines using C2-9 or C1-5 curvilinear probes. All data were anonymized at the point of collection using specifically designed video capture software that blanks out the patient identifiable information on the screen recording.

The enrollment of participants, clinical data collection, and the ultrasound scans (CALOPUS and routine clinical scans) were performed by a dedicated clinical research team of doctors, nurses, and sonologists. All women were eligible if they were able to provide informed consent, were aged ≥18 years, and had (or were attending for) a scan between 11 and 14 weeks of pregnancy for gestational age assessment based on fetal crown-rump length (CRL).

In India, participants were recruited from the ongoing interdisciplinary Group for Advanced Research on BirtH outcomes–Department of Biotechnology India Initiative (GARBH-Ini) cohort [12]. This is a prospective observational cohort of pregnant women enrolled before 20 weeks of gestation. All pregnant women were followed up during pregnancy with ultrasound scans at 18 to 20, 30 to 32, and 35 to 37 weeks of gestation, and birth outcomes were determined. At Oxford, women attending the antenatal ultrasound department of the John Radcliffe Hospital were invited to enroll throughout pregnancy from 11+0 weeks of gestation. Consecutive recruitment was up to a weekly quota of 20 scans per week and could be targeted to better focused recruitment of women in specific gestational age windows.

As the incidence of breech presentation reduces to approximately 3% to 4% at term [13], the anticipated number of scans recorded of noncephalic presenting fetuses would inevitably be small, even in a large data set. As machine learning models require a degree of balance in the training data set, to ensure a higher proportion of ultrasound scans in women with noncephalic fetal presentation, women in the Indian cohort with a noncephalic fetus at the 30 to 32 or 35 to 37 weeks scan received additional scans every 15 days until the presentation became cephalic or until delivery.

### Ethics Approval

In India, ethics approval was obtained from the institutional ethics committees of the Translational Health Science and Technology Institute, Faridabad, India, THS/1.8.1/(71), dated August 26, 2019, and Gurugram Civil Hospital, Haryana, India, GHG/IEC letter, dated September 3, 2019. Written informed consent was obtained from all study participants by the study nurses under the supervision of research officers. For illiterate women, the details of the study were explained in the presence of a literate impartial witness. Verbal consent and thumb impressions were taken from the participants along with the signature of the witnesses.

In the United Kingdom, approval from the West of Scotland Research Ethics Committee 5 (reference 18/WS/0051) was obtained, along with approvals from the Health Research Authority and Oxford University Hospitals. Trained research midwives or the sonologists themselves completed the consent process with participants, and written informed consent was obtained.

### Defining the CALOPUS Scan Acquisition Protocol

Due to the exploratory nature of this collaboration, the protocol for scan acquisition developed over time. In 2016, Abuhamad et al [11] published a clinical study validating the feasibility and accuracy of a 6-step approach to performing a focused basic obstetric ultrasound. Each step corresponds to an integral part of prenatal care and helps identify women at high risk of obstetric complications. For example, in a setting with poor health care and transport infrastructure, it would be ill-advised for a woman to attempt a vaginal breech birth or delivery of twins without access to comprehensive emergency obstetric care facilities. Similarly, a woman with known major placenta previa should be delivered by cesarean section and not attempt vaginal birth. The purpose of this 6-step approach was to provide a simple framework that could be readily taught to health care providers with limited scanning experience. A series of 5 simple steps allows the evaluation of fetal presentation, presence of cardiac activity (fetal viability), presence of multiple pregnancies, determination of placental location, and amniotic fluid measurement. The sixth step is a standard clinical freehand scan for fetal biometry.

This 6-step approach was adopted as the initial scanning protocol for the CALOPUS. However, although a protocol for human point-of-care ultrasound benefits from sequential decision-making, it contains overlap and redundant information, that is, each step contains information relevant to more than one of the six aims. A hypothesis of this study is that automation offers the opportunity to optimize the scanning protocol to reduce redundancy in overlapping scan sweeps. To test this hypothesis, we conducted an initial analysis of 470 videos from the first 80 participants. All women had viable second or third trimester pregnancies (gestational age range from 19+3 to 40+0 weeks).

### Clinical Protocol Refinement to One Optimized for Machine Learning

On the basis of this, the original 6-step approach was modified into the CALOPUS ultrasound protocol (Figure 1 and Multimedia Appendix 2). Steps 1 and 5 of the original approach were removed to streamline the acquisition process. Hence, the original step 2 became the new step 1 of the CALOPUS ultrasound protocol, as it consistently confirmed both fetal presentation and viability.

Steps 2 and 3 of the CALOPUS ultrasound protocol were from the original 6-step approach and aim to identify multiple pregnancies and quantify amniotic fluid**.** We used maximum vertical pool (MVP) depth to assess amniotic fluid volume (rather than the amniotic fluid index due to its simplicity), as neither have been demonstrated to be superior in predicting adverse outcome. Another reason to use MVP depth was that amniotic fluid index results in a higher number of diagnoses of oligohydramnios than MVP depth, which in turn may result in an increased number of inductions of labor and cesarean sections but without benefits to perinatal outcomes [14,15].

Even in combination with other steps, step 4 of the original 6-step approach was found to be inadequate for determining the relationship between the placenta and the cervix, because the cervix is not always directly behind the pubic symphysis. This is important, as clinically, a low-lying placenta is ruled out by measuring the distance from the placental margin to the internal cervical os. It is widely accepted that an anterior placenta will not impede vaginal delivery if it is more than 10 mm from the internal os during the second trimester. It has been recommended that the posterior placenta should be >15.5 mm from the internal os in the second trimester [16]. Hence, a new step (step 5 of the CALOPUS ultrasound protocol) was introduced to improve visualization of the uterine lower segment and cervix and to reduce the number of false positive cases (where the placenta is not low-lying but is suspected to be so). This was a U-shaped sweep from the right to the left iliac fossa, along the maternal pelvic brim.

The final step of the CALOPUS ultrasound protocol (step 6) is a validation step to measure the CRL if <14 weeks gestation or the head circumference, transcerebellar diameter, biparietal diameter, abdominal circumference, femur length, and the deepest pool of amniotic fluid if ≥14 weeks (CRL>84 mm [17]) according to previously described ultrasound methodology [18-20]. This was done so that any measurable biometry planes were identified from steps 1 to 5 of the CALOPUS ultrasound protocol, and biometric measurement values were compared with the paired standard clinical ultrasound. The methodology for measuring fetal biometry and amniotic fluid remained constant across all acquisition protocol iterations, with the use of a standardized protocol from international standards [20,21] to ensure the quality control of the images obtained.

### Statistical Justification

A pilot study was conducted to calculate the sample size required to answer the following questions:

1. Does the addition of step 5 of the CALOPUS ultrasound protocol help to visualize the cervix more than step 4 of the CALOPUS ultrasound protocol alone?
2. Does the fullness of the bladder have more of an impact on visualization of the cervix than the addition of step 5 of the CALOPUS ultrasound protocol?
3. Is the addition of step 5 of the CALOPUS ultrasound protocol clinically significant?

The pilot study focused on women between 18 and 22 weeks of gestation, because at this gestation, it should be possible to confirm placental location with transabdominal scanning alone. At more advanced gestations, a transvaginal scan is often necessary to check the distance between the placental margin and cervix, particularly when the placenta is posterior and within the lower segment of the uterus. The pilot study was a paired cohort study in which data were analyzed using McNemar test (a 2-sample paired-proportions test) performed in STATA (version 16.1). A total of 69 women underwent both steps 4 and 5 of the CALOPUS ultrasound protocol. The cervix was defined as having been seen if part of the endocervical canal was visible, and the bladder was well filled if it contained >200 mL of urine as defined by a cuboid volumetric calculation. This recommendation for bladder fullness is based on guidance for placental imaging, because a full bladder stretches out the lower segment to provide a more realistic idea of the relationship between the placental edge and the cervix or any previous cesarean scar [22].

The sample sizes were calculated using type 1 error rate of 0.05 and 90% power. A sample size of 212 scans was needed to detect the differences observed in the pilot study between the addition of steps 5 and 4 of the CALOPUS ultrasound protocol alone or the contribution of a full bladder in correctly diagnosing the placental location, compared with a freehand scan gold standard.

### Data Processing

The ultrasound machine video output port was split at both sites. One port was connected to an image-acquisition hardware installed on a desktop computer. Data collection software was implemented to control the image-acquisition hardware and to collect the ultrasound videos in real time, without affecting the scanning procedure. The recordings were saved locally on a desktop computer.

### Data Quality Checks and Storage

Scans underwent manual quality checking for the following reasons:

- To assess whether all the scans have synced properly from the clinical site
- To assess if the ultrasound signal had been successfully captured in the video
- To assess if any steps or sweeps were missing from the scan directory
- To record the duration of the video files
- To undertake a basic visual check of video quality (eg, image corruption or distortion).

The scans were then transferred to the server for annotation and subsequent analysis at each site.

### Annotation Protocol

The annotation of key anatomical structures in the CALOPUS ultrasound protocol video sweeps are used alongside the video as the training input to the machine learning algorithms.

Initial manual annotations were performed by a single annotator who placed bounding boxes around the structures of interest using a VATIC backend [23] and a self-designed XML administrator web page on the data server desktop. The annotation tool was subsequently changed to CVAT [24] to increase functionality by including segmentation and point annotation with more attributes. Irrespective of the tool used, frames were not annotated where there was significant motion artifact.

### Quality Assurance of Annotations

Acquiring the large number of annotations required for machine learning models can be prohibitively slow using a single annotator. In addition, a single annotator may introduce annotation bias. We used a team of 5 annotators in the United Kingdom and India to increase the rate of annotation and to reduce bias, but this posed a new challenge: How can we quantify the acceptable variation between annotators to ensure an achievable standard for the annotation team?

The annotators were sonologists with experience in performing antenatal ultrasound scans. A standard operating procedure was developed (Multimedia Appendix 3), and several metrics were selected to enable comparison between annotators. A standardization exercise was undertaken by the first 2 members of the annotation team, using annotations of 9 recordings of step 1 (6712 frames). These were annotated on a frame-by-frame basis, placing bounding boxes around 11 anatomical features of interest: the fetal head; cerebellum; heart; spine; abdomen; pelvis; stomach and femur; and the maternal bladder, amniotic fluid, and placenta. Following the standardization of the 9 videos, annotators were then assessed on further 20 videos to ensure that their annotations were in line with the standard operating procedure of the annotation. For ongoing quality assurance, every tenth annotation was repeated by a second annotator to ensure consistency was maintained over time.

To guide the expected levels of agreement required for new annotators to achieve and provide an expected standard for ongoing annotation quality assurance, we conducted a baseline intra- and interannotator agreement study. Each of the 2 annotators annotated all the 18,717 frames from 20 videos for the 11 anatomical features described earlier. They repeated all annotations 2 weeks later to allow calculation of both intra- and interannotator agreement.

We developed a code to assess the agreement between sets of annotations available on GitHub [25]. The metrics are calculated as follows:

- Partial match: a frame was denoted as a partial match if >50% of the labels were the same between 2 different annotations.
- Exact match: a frame was denoted as an exact match if the bounding boxes drawn in each frame were the same between 2 different annotations divided by the total number of frames.
- Match per label: closely related to the exact match for an individual anatomical feature, with the denominator as the total number of frames with a given anatomy label by either of the annotators.
- Bounding box intersection over union: calculated for 2 bounding box annotations of the same feature on the same frame. The total area of the bounding box overlap (the intersection) was divided by the area of union between the 2 annotations (total area of both annotators’ bounding boxes for any feature minus the area of intersection).

## Results

### Participants

As of June 2022, a total of 5661 participants have been recruited into the CALOPUS study. After excluding 90 scans following manual quality checking of the scans, 5571 (98.41%) participants remained. Table 1 shows the number of scans acquired at different gestational ages through the iterations of the CALOPUS protocol.

**Table 1:** Ultrasound data collected as part of the Computer-Assisted Low-Cost Point-of-Care UltraSound (CALOPUS) study.

Gestational age (weeks)	Six-step approach (n=431), n (%)	CALOPUS ultrasound protocol (n=5140), n (%)
<14	4 (0.9)	909 (17.7)
14+0 to 17+6	1 (0.2)	435 (8.5)
18+0 to 24+6	265 (61.5)	1485 (28.9)
25+0 to 29+6	7 (1.6)	354 (6.9)
30+0 to 34+6	69 (16)	1022 (19.9)
≥35+0	85 (19.7)	935 (18.2)

### Defining the CALOPUS Protocol

Step 1 of the 6-step approach confirmed presentation in only 60% (46/77) of the cases, whereas step 2 (designed to detect the fetal heart) was able to do so in 99% of the cases (79/80; the single failure was for a twin pregnancy). Conversely, step 2 was able to confirm viability in 81% (62/77) of the videos, and analysis of one or more additional steps was needed to determine fetal heart activity, which was feasible in 99% (79/80) of the participants. Furthermore, steps 3.2 and 5 of the 6-step approach sweep across the same parts of the maternal abdomen, so that a computational algorithm could measure pools of amniotic fluid in step 3.2 rather than requiring a repeat (step 5). Placental location was the most difficult component of the scan to assess. Steps 3 and 4 combined located the placenta in 86% (69/80) of the cases. Ruling out a low-lying placenta was particularly difficult for posterior placentas or those within the lower segment where the cervix could not be visualized.

This initial analysis concluded that a revised protocol for automated decision-making algorithms should be designed to reduce the number of steps owing to redundancy in information obtained. However, more than one step may be needed to achieve each objective, in particular for the determination of placenta location.

We subsequently analyzed 212 scans to assess the impact of step 5 of the CALOPUS ultrasound protocol and bladder filling (Table 2). Our results showed that the cervix was visualized 4-fold more frequently when step 5 of the CALOPUS ultrasound protocol was included as well as step 4 (n=120), compared to 29 times when using step 4 of the CALOPUS ultrasound protocol alone. The bladder was well filled (>200 mL) in 30.7% (65/212) of the women. The cervix was visualized in 91% (59/65) of the cases where the bladder was well filled and both steps 4 and 5 of the CALOPUS ultrasound protocol were assessed, compared with the cervix seen in 28% (18/65) of the scans where only step 4 of the CALOPUS ultrasound protocol was considered. This demonstrates that both bladder fullness and the addition of step 5 of the CALOPUS ultrasound protocol have a considerable impact on the frequency at which the cervix is visualized. The addition of step 5 of the CALOPUS ultrasound protocol is more significant than bladder filling alone.

**Table 2:** The impact of bladder filling and different steps on visualization of the cervix and classification of placental location.

				Bladder well filled (n=65), n (%)		Bladder poorly filled (n=147), n (%)
**Visualization of the cervix**						
	**Step 4 alone**					
		Seen (n=29, 13.7%)	18 (28)		11 (7)	
		Unseen (n=183, 86.3%)	47 (72)		136 (93)	
	**Steps 4 and 5**					
		Seen (n=120, 56.6%)	59 (91)		61 (41)	
		Unseen (n=92, 43.3%)	6 (9)		86 (59)	
**Classification of placental location**						
	**Step 4 alone**					
		Correct (n=165, 77.8%)	53 (82)		112 (76)	
		Incorrect (n=47, 22.2%)	12 (18)		35 (24)	
	**Steps 4 and 5**					
		Correct (n=189, 89.2%)	61 (94)		128 (87)	
		Incorrect (n=23, 10.8%)	4 (6)		19 (13)	

It should be noted that it is not always necessary to visualize the cervix to correctly identify whether a placenta is low-lying (eg, when the placenta is at the uterine fundus). Therefore, we examined whether the addition of step 5 of the CALOPUS ultrasound protocol was clinically beneficial by increasing the correct classification of low-lying placental position compared with a standard ultrasound examination. A protocol including both steps 4 and 5 of the CALOPUS ultrasound protocol correctly classified placentas as low-lying or not in 89% (189/212) of the instances compared to 78% (65/212) with step 4 of the CALOPUS ultrasound protocol alone.

These data indicate that the CALOPUS ultrasound protocol should include a low pelvic brim sweep (step 5). Women should be scanned with a full bladder to optimize visualization of the lower segment and assist in the classification of placental location.

### Annotations

Manual annotation is very time-intensive: a video clip lasting 20 to 30 seconds typically takes approximately 40 minutes to fully annotate with bounding boxes. This is because of the time taken to change the size and position of the bounding boxes in response to the probe and fetal movements (despite using an interpolative setting from one frame to the next). The total number of frames recorded for each participant is approximately 4500 (30 frames per second), and so routinely, only 1 or 2 video clips per participant were fully annotated with bounding boxes around the structures of interest, with other videos having frame-level anatomy labels. At the time of publication, 1,541,751 label annotations were made. Overall, 1,026,744 (66.6%) annotations were bounding box annotations around the features of interest, 7788 (0.5%) were segmentations, and 507,219 (32.9%) were labels assigned to frames.

As opposed to bounding box annotation, frame labeling significantly reduces the annotation time but comes at the expense of having no information about the location of a structure within a frame. Table 3 lists the number of videos and frames labeled.

**Table 3:** Number of video annotations in the Computer-Assisted Low-Cost Point-of-Care UltraSound study.

	Videos (n=1057), n (%)	Frames (n=586,253), n (%)
Step 1	446 (42.2)	254,095 (43.3)
Step 2	161 (15.2)	114,958 (19.6)
Step 3	163 (15.4)	67,633 (11.5)
Step 4	164 (15.5)	103,269 (17.6)
Step 5	123 (11.6)	46,298 (7.9)

### Quality Assurance of Annotations

The levels of agreement between annotators and their repeated annotations over 18,717 frames are listed in Table 4. Annotator 2 demonstrated greater reproducibility in annotation than annotator 1, but the same patterns were observed throughout: partial match percentages were higher than for exact label match, and the reproducibility of an annotator’s own annotations was greater than the agreement between 2 different annotators. The pooled κ values for match per label demonstrated an excellent level of agreement both between annotators and for repeat annotations, and the bounding box intersection over union >50% was high.

**Table 4:** Baseline intra- and interannotator agreement in the Computer-Assisted Low-Cost Point-of-Care UltraSound study.

	Intra-annotator agreement			Observed interannotator agreement
	Annotator 1	Annotator 2		
Partial match (%)	98.1	98.3	94.5	
Exact match (%)	84	91.1	71.4	
Match per label (%)	91.8	95.7	83.1	
Pooled κ	0.93	0.96	0.85	
Bounding box overlap >50% (%)	94.8	97.1	93.4	

## Discussion

### Principal Findings

The CALOPUS study aims to develop and evaluate simple-to-use clinical protocols and machine learning–based decision-making tools for nonexpert users that are suitable for use in underserved regions. In this paper, we present the methods used to collect clinically relevant, detailed, repeatable, and quality-assessed data for the development of machine learning algorithms for obstetric ultrasound.

### Strengths and Limitations

This study has several strengths and uses a multidisciplinary clinical engineering team approach. The prospective nature of data collection allows meticulous quality assurance, which is not possible with retrospective collection of existing clinical data. As a prospective study, it also allows us to collect targeted data for the purpose of machine learning on ultrasound videos, which is not routinely collected in most settings. Preliminary investigation of a machine learning model for automatic detection of breech presentation, built and validated on these data has been described [26], and current work is ongoing to improve this; we are also developing machine learning model for automatic assessment of low-lying placentas [27] and another for preventing fetal sex determination (mandated in some settings), achieved by automated recognition and obscuring of the fetal pelvic region. We also used a subset of the CALOPUS data and annotations from the Automatic Amniotic Fluid Measurement and Analysis From Ultrasound Video Ultrasound Challenge: Automatic amniotic fluid measurement and analysis from ultrasound videos [28]

We believe that the dual site and international nature of this collaboration increases external validity and enrollment. Nevertheless, this is at the expense of adding complexity to the setup of the study sites and standardization processes. Challenges included training of staff in the use of the ultrasound protocol and annotation of data, which had to be performed remotely. This increased the risk of undesirable changes. Fortnightly meetings between the data acquisition and annotation teams were undertaken to ensure that logistical and technical issues could be identified and corrected promptly and that feedback regarding scanning and annotation variations could be discussed. Despite this, when recruitment speed exceeds annotation speed, problems with data collection may not become evident for some time, as was the case on one occasion. We have now introduced methods to automate some of the quality checks, such as sweep duration to give advice about scanning speed and to identify frames “jumping,” which may only be noticed when watching videos at much slower playback speeds.

An important limitation of our study was the use of high-cost ultrasound equipment by expert sonologists. Although our ultimate aim is to develop low-cost ultrasound solutions, there were several reasons why we opted to use a GE Voluson E8 machine during this phase of the study. First, the scan images produced are of high quality. We believe this is a reasonable first step when building machine learning algorithms by training models with high-quality images initially and then introducing lower-quality images later on. Domain adaptation techniques are available for this purpose, which allow parameter refinement from a model built with large data in one domain (in our case, a high-cost ultrasound machine) to a second domain with a small amount of data (in our case, a low-cost ultrasound machine). Second, both clinical sites had GE Voluson E8 machines, which simplified the initiation of the study along with routine clinical scanning. Having demonstrated the feasibility of higher cost systems, we will then translate the models we develop into clinically useful tools to be used by minimally trained personnel with low-cost, handheld ultrasound technology.

Despite being a collaborative project, there have been limitations from the respective ethics committees regarding what data can be shared between sites. Thus, although 1 in 10 scans can be shared for quality assurance purposes, the study design of the machine learning model development and testing was affected. One solution is to build single-site models that use a second site for testing [27]. Another is the use of transfer learning, whereby a model is built at one site and refined using data at the second site. Federated learning is also being considered, where a model is trained on data available at each site separately, and once site-specific models are built, model weights are combined to give a final model [29]. This emerging approach is beneficial as the transfer of original data is not required; rather, encoded weights are shared. We believe this is likely to benefit wider use for multisite machine learning–based imaging research in the future.

### Challenges and Further Scope

We observed a lack of universal tools for annotating ultrasound videos. It would be advantageous for there to be open source software that truly caters to the annotation of medical images and videos by accepting multiple file types, enabling measurements to be taken, and allowing frame labeling, bounding box annotation, and segmentation. Currently, several different tools need to be used to perform these tasks.

For most human-executed tasks, reproducibility between ultrasound practitioners is lower than that within the same practitioner [30]. Widely used guidelines exist for the standardized acquisition of ultrasound images in clinical practice [31-33]. We are not aware of any standards for manual annotation and were compelled to develop definitions for labeling different anatomies owing to the complexity of fetal position and movements. The agreement between sonographers assessing a single frame is not 100%, so even a lower agreement is expected when annotating a video consisting of hundreds of frames. The assessment of interannotator agreement is also important in identifying the best level of agreement that could be expected between a human observer and a machine learning model. As manual annotations of anatomical planes provide the only way for the clinical ground truth of anatomical standards to be demarcated, it would be unreasonable to aim for 100% agreement when experienced annotators cannot consistently reach this target of agreement.

Finally, it is worth reflecting on the challenges of interdisciplinary research in this emerging research area. Clinical assessment of CALOPUS videos is novel; therefore, explicitly translating this into a machine learning algorithm input is exploratory in nature. Interdisciplinary research explores the rules needed to assess diagnostic information from both the human and artificial intelligence perspectives. Engineers and clinicians need to work together to understand both perspectives and benefit from regular and clear communication to discuss study design, meet each other’s data requirements, and understand differing perspectives about important research questions. Our clinical team has found this an exciting area to work in as it allows a better understanding of clinical decision-making processes when these are explicitly verbalized, whereas the engineering team has found working on real-world clinical problems highly rewarding, as these show interesting, unique challenges not commonly encountered elsewhere.

### Conclusions

The CALOPUS study is a unique study that uses obstetric ultrasound videos and annotations from pregnancies dated from 11 weeks and followed up until birth using novel ultrasound and annotation protocols. The data from this study are being used to develop and test several different machine learning algorithms to address key clinical diagnostic questions for obstetric risk management. We also highlight some of the challenges and potential solutions to interdisciplinary international collaboration.

## Appendix

Multimedia Appendix 1 Members of the Computer-Assisted Low-Cost Point-of-Care UltraSound study group.

Multimedia Appendix 2 The Computer-Assisted Low-Cost Point-of-Care UltraSound protocol.

Multimedia Appendix 3 The Computer-Assisted Low-Cost Point-of-Care UltraSound annotation protocol.

## Abbreviations

CALOPUS: Computer-Assisted Low-Cost Point-of-Care UltraSound
CRL: crown-rump length
GARBH-Ini: Group for Advanced Research on BirtH outcomes–Department of Biotechnology India Initiative
MVP: maximum vertical pool

## Data Availability

The data sets generated and those analyzed during this study are not publicly available owing to ethical constraints. The authors have freely shared a link to assess the agreement between sets of annotations available on GitHub [25].
