# Supplementary material for: Developing Clinical Artificial Intelligence for Obstetric Ultrasound to Improve Access in Underserved Regions: Protocol for a Computer-Assisted Low-Cost Point-of-Care UltraSound (CALOPUS) Study
Source: JMIR Res Protoc. 2022 Sep 1;11(9):e37374. doi: 10.2196/37374 (PMC9478819; doi:10.2196/37374)
Supplement: Multimedia Appendix 1 [file resprot_v11i9e37374_app1.docx]

# Additional file 1

## Members of the CALOPUS Study Group

| Professor Alison Noble | [alison.noble@eng.ox.ac.uk](mailto:alison.noble@eng.ox.ac.uk) | UK |
| --- | --- | --- |
| Professor Aris Papageorghiou | [aris.papageorghiou@wrh.ox.ac.uk](mailto:aris.papageorghiou@wrh.ox.ac.uk) | UK |
| Professor Shinjini Bhatnagar | [shinjini.bhatnagar@thsti.res.in](mailto:shinjini.bhatnagar@thsti.res.in) | India |
| The interdisciplinary Group for Advanced Research on BirtH outcomes -Department of Biotechnology India Initiative (GARBH-Ini) | [garbhinistudy@thsti.res.in](mailto:garbhinistudy@thsti.res.in) | India |
| Mr Varun Chandramohan | [c.varun@thsti.res.in](mailto:c.varun@thsti.res.in) | India |
| Dr Qingchao Chen | [qingchao.chen@eng.ox.ac.uk](mailto:qingchao.chen@eng.ox.ac.uk) | UK |
| Ms Ali Chevassut | [alison.chevassut@wrh.ox.ac.uk](mailto:alison.chevassut@wrh.ox.ac.uk) | UK |
| Ms Rachel Craik | [Rachel.craik@wrh.ox.ac.uk](mailto:Rachel.craik@wrh.ox.ac.uk) | UK |
| Dr Bapu Koundinya Desiraju | [bapukoundinyadesiraju@thsti.res.in](mailto:bapukoundinyadesiraju@thsti.res.in) | India |
| Mr Sumeet Dhariwal | [sumeet.dhariwal@eng.ox.ac.uk](mailto:sumeet.dhariwal@eng.ox.ac.uk) | UK |
| Mr Alexander Gleed | [alexander.gleed@balliol.ox.ac.uk](mailto:alexander.gleed@balliol.ox.ac.uk) | UK |
| Dr James Jackman | [james.jackman@eng.ox.ac.uk](mailto:james.jackman@eng.ox.ac.uk) | UK |
| Dr Ashok Khurana | [ashokkhurana@ashokkhurana.com](mailto:ashokkhurana@ashokkhurana.com) | India |
| Ms Vidhya Krishnamoorthy | [vidhya.krishnamoorthy@thsti.r](mailto:vidhya.krishnamoorthy@thsti.r) | India |
| Mr Divyanshu Mishra | [mdivyanshu.ai@gmail.com](mailto:mdivyanshu.ai@gmail.com) | India |
| Mr Pramit Saha | [pramit.saha@eng.ox.ac.uk](mailto:pramit.saha@eng.ox.ac.uk) | UK |
| Dr Alice Self | [alice.self@wrh.ox.ac.uk](mailto:alice.self@wrh.ox.ac.uk) | UK |
| Dr Ramachandran Thiruvengadam | [ramachandran@thsti.res.in](mailto:ramachandran@thsti.res.in) | India |
| Dr Nitya Wadhwa | [nitya.wadhwa@thsti.res.in](mailto:nitya.wadhwa@thsti.res.in) | India |
| Dr Elizabeth Wilden | [elizabeth.wilden@eng.ox.ac.uk](mailto:elizabeth.wilden@eng.ox.ac.uk) | UK |
