# Supplementary material for: Developing Clinical Artificial Intelligence for Obstetric Ultrasound to Improve Access in Underserved Regions: Protocol for a Computer-Assisted Low-Cost Point-of-Care UltraSound (CALOPUS) Study
Source: JMIR Res Protoc. 2022 Sep 1;11(9):e37374. doi: 10.2196/37374 (PMC9478819; doi:10.2196/37374)
Supplement: Multimedia Appendix 2 [file resprot_v11i9e37374_app2.docx]

# Additional file 2

## The CALOPUS Ultrasound Protocol

**Preparation and set up**

Apply a liberal amount of gel to the whole maternal abdomen and ensure that the dimple of the probe is always pointing towards the maternal right or maternal head so that her bladder will always be on the right side of the screen as it’s looked at. Next, adjust the depth and focus so that the internal os of the cervix can be visualised and there is adequate depth to scan the entirety of the uterine depth at the level of the umbilicus. It is recommended that a woman is scanned with a full bladder.

**Step 1**

- Place the transducer transversely on the lower abdomen just above the symphysis pubis at 90⁰ to the floor.
- Move the transducer up towards the mid-abdomen towards the umbilicus, keeping the transducer in the transverse orientation and 90⁰ to the floor.
- Then, without lifting up the probe, move the transducer to the (woman’s) right side of the abdomen from the umbilicus whilst maintaining a transverse orientation.
- Then move the transducer from the umbilicus to the left side of the abdomen without lifting off the probe and still maintaining the transverse orientation.

**Step 2**

- Place the transducer in the transverse orientation on the lower abdomen by the right superior iliac spine.
- Scan the uterine cavity in a straight line up to the woman’s ribs along track 1
- Repeat from the symphysis towards the participant’s head along tracks 2 and the left iliac spine to lower left ribs along track 3.
- The transducer’s orientation must stay transverse and perpendicular to the floor and the sweeps should be parallel to each other.
- Tracks 1 and 3 should be no more lateral than the woman’s anterior superior iliac spine.

**Step 3**

- Place the transducer in the upper right abdomen in the sagittal position and perpendicular orientation to the floor.
- Move the transducer from the maternal right to maternal left along tracks 1 and 2 maintaining its orientation

**Step 4**

- Place the transducer in the woman’s right upper quadrant, in a sagittal orientation.
- Scan caudally towards the right iliac crest along track 1.
- Repeat along tracks 2 and 3 by moving the probe to start just below the xiphisternum (2) and then the left upper quadrant (3).
- The transducer’s sagittal orientation must stay perpendicular to the floor.

**Step 5**

- Place the probe in the sagittal orientation in the right iliac fossa next to the right anterior iliac spine.
- Scan the uterine cavity from the right iliac fossa to the left along the pelvic brim following the track between 1 and 2.
- Maintain the transducer’s sagittal orientation perpendicular to the floor
