# Supplementary material for: Developing Clinical Artificial Intelligence for Obstetric Ultrasound to Improve Access in Underserved Regions: Protocol for a Computer-Assisted Low-Cost Point-of-Care UltraSound (CALOPUS) Study
Source: JMIR Res Protoc. 2022 Sep 1;11(9):e37374. doi: 10.2196/37374 (PMC9478819; doi:10.2196/37374)
Supplement: Multimedia Appendix 3 [file resprot_v11i9e37374_app3.docx]

# Additional file 3

## The CALOPUS annotation protocol

Manual annotation was performed using the CVAT software tool, versions 1.2 and 1.3 which is available at <https://github.com/openvinotoolkit/cvat>. The instructions below refer to using this version of the tool.

## Labelling procedure

1. First watch or scroll through entire video to orientate yourself to the baby’s position and which anatomical structures you can see within the video.
2. You may wish to annotate smaller structures first as they get easily occluded by multiple labels in one frame.
   - ***Make sure that you don’t draw a box around a structure unless it is the first frame that it is visible in within the video.***
3. Draw a box around the area of interest as described above. The default label is none, so ensure that you change this to the relevant structure.
   - ***It is more time efficient to make the boxes a few millimetres too big so that you don’t have to move it or change the box size so frequently.***
4. Skip forward through frames either using the keyboard or clicking on the arrow buttons adjusting the size and moving the bounding box as needed.
   - ***Be aware that some structures, such as the placenta and amniotic fluid, change size very quickly and the computer’s interpolation can’t keep up, so always go back and check previous frames – ensure the structure remains within the bounding box as the size or position of the box changes.***
5. When a structure is no longer visible click on the
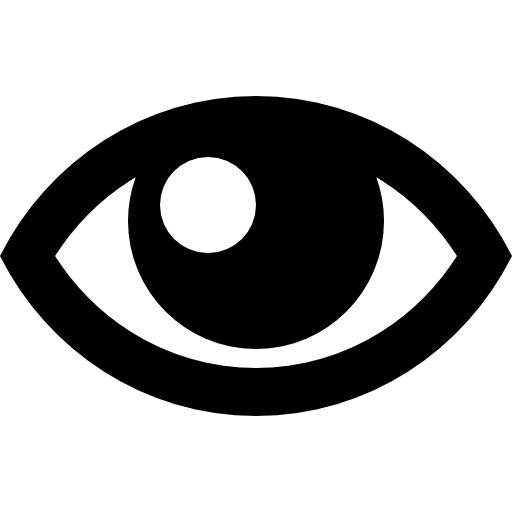
 (version 1.2) or
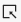
 or “switch to outside property [O]” (version 1.3) and the box will disappear from that frame onwards.
6.
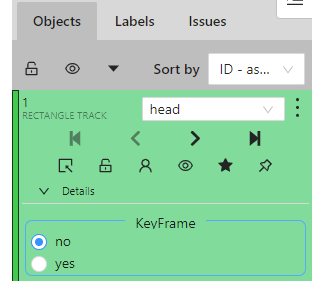

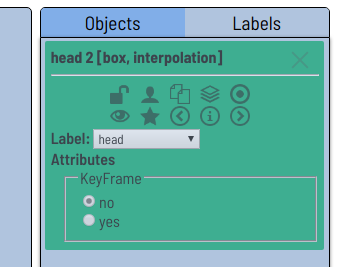
Structures that are measured for biometry include the head, cerebellum, abdomen and femur. We want to train algorithms to take these measurements from the best possible frames in the sweeps recorded. If you see any frames similar to the examples below select the ‘yes’ where it says ‘KeyFrame’ under **Attributes** (version 1.2) or **Details** (version 1.3). Selecting this will work on all subsequent frames until you select ‘no’ again. The default option is always ‘no’.
   - ***Images do not need to be “textbook”. The aim is to create a bank of images that approximate perfect biometry planes by including “key frames” in the vicinity of textbook images.***
   - Head circumference frames should have a complete oval skull outlined, and the cavum septum pellucidum should be visible in the anterior third.

***
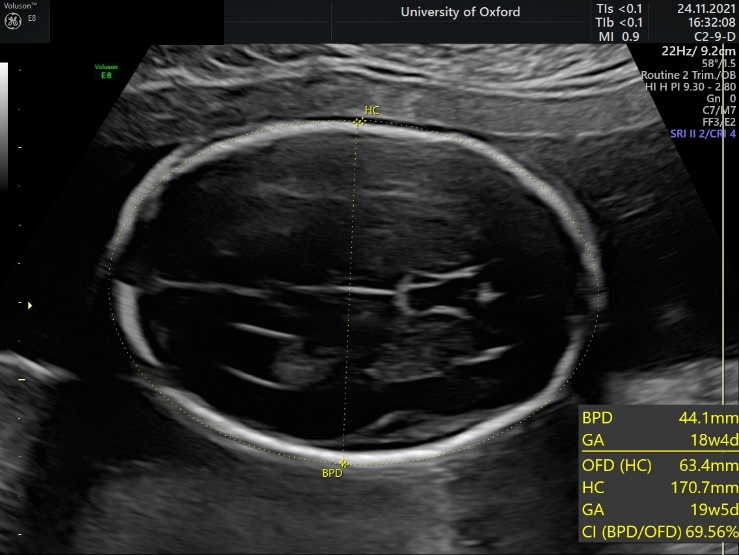
***

- - ***
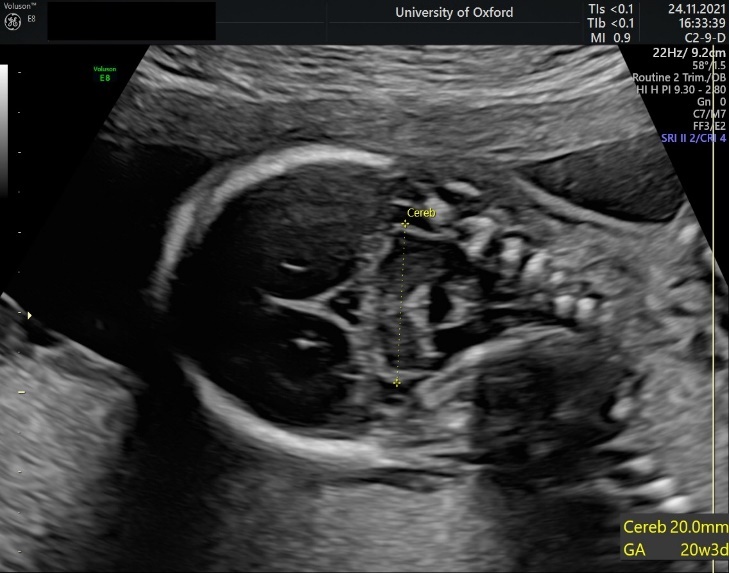
***Cerebellum –symmetrical and viewed either in a transverse or coronal section.
  - Abdominal circumference should be round with straight ribs, with the stomach bubble or umbilical vein showing. Kidneys should NOT be visible.

******

- - Femur length – full length visible

******

1. Repeat for all structures identifiable in the video and then click on **open menu** and **save work** to save the labels.
2. If at any point you need to take a break, save your work first. Having done so, you can return to the home screen of the CVAT app by clicking on the back tab of your web browser.

## Definitions of each structure

Remember that structures may be seen in a coronal, sagittal, axial or oblique orientation but still need to be labelled.

In general it is better to have a smaller, “cleaner” dataset than a larger “noisier” one for machine learning purposes, BUT this relates to the number of frames labelled not to the size of a bounding box. It is better that boxes around fetal parts are too big rather than too small. The only label that doesn’t need so much generosity is amniotic fluid as there’s plenty of it!

### Abdomen

**Range:** From level of umbilicus to diaphragm (just below the heart). If an oblique view is seen, boxes *should* overlap with the pelvis or heart. Generally, there wouldn’t be much abdomen in view where the ribs are visible but these may need to be included in your box in order to include abdomen elsewhere when the plane is diagonal to the long axis of the fetus, e.g. if liver visible and clearly distinguishable from lungs in the thoracic cavity.

**Key frame:** only the transverse section near the abdominal circumference measurement plane, with at least the stomach bubble or umbilical vein visible.

### Amniotic fluid

Any clear pocket of AF should be labelled. **If the edges of the pocket are very blurry it is best not to label it**. The AF labels and bladder labels should not occur over the same space as this will confuse the machine learning.

When there is a large pocket on one side of the fetus and then a new one appears on the other side, it is better to draw a new box for the second pool of AF.

### Cerebellum

**Range:** May only see one cerebellar hemisphere at a time.

**Key frame:** When both hemispheres are visible for measuring, in either coronal or axial views. Only label if outline of cerebellar hemispheres are clearly visible.

### Head (includes face)

**Range:** Any part of the head or face from the chin, lips and nose to the tip of the skull.

**Key frames:** only the views closest to a good head circumference place with the CSP visible should be marked as a key frame.

### Heart

Draw a big enough box to include the whole myocardium. Greater vessels/outflow tracts do not need to be included. Only label if clearly visible and not if it was there, or is about to appear again, Pulsations in a black haze are not sufficient to label the heart – it must be clearly demarcated.

### Femur

**Range:** Any part of the femur - the full length will rarely be seen. Easily confused with the humerus, especially later in pregnancy, so if in doubt, don’t label it. Only label if clear, bright white bone.

**Key frame:** when the whole shaft of femur is visible.

### Pelvis

This is being labelled to help identify the region that needs to be obscured to avoid sex determination. To ensure this it has been decided to label the Pelvis as any level below the cord insertion and up to the point at which the buttocks are no longer visible. This includes the iliac crest or fetal bladder. Please label down to the point at which the buttocks are no longer visible as external genitalia can be seen at any point and we want the genitalia included in the pelvis bounding box. Start the label at the proximal end of the femur. It is expected that abdomen and pelvis boxes will overlap so please ensure they do if both seen in the same frame. Also, it is expected the pelvis box will overlap the top of the thighs so it may overlap femur boxes also.

### Placenta

Label the entire placenta, but be sure you are not labelling a contraction of uterine muscle that can imitate a lobe of placenta. If in doubt, it is best not to label it.

### Spine

Label in coronal, sagittal and axial views. The cervical spine will often overlap with the head box. If occluded by scapulae or pelvis then mark it as no longer visible and draw a new box when it reappears.

### Stomach bubble

Almost all babies will be situs solitus and so you would expect the stomach bubble to be on the same side as the heart. It can look similar to the gall bladder and some intrahepatic vessels so be careful with labelling. **It should always be fully within an abdomen box.**

### Bladder (maternal only)

This label is to help the algorithm learn how to distinguish AF from the bladder and also to help locate the placenta in relation to the lower edge of the maternal bladder. Only label where it has a clearly definable bladder shape; do not label if you are not sure if you are seeing bladder or AF.

### Cervix (maternal only)

Only in sagittal views (Not step 1 or 2.1). Important for placental localisation but often difficult to identify, especially if the bladder is empty or the fetus is low-lying. The cervical canal should be a slightly darker line and if seen constitutes a key frame. If unsure, it is best not to label it.

### Vagina (maternal only)

Only in sagittal views (Not step 1 or 2.1). A hyperechoic line will be seen where the anterior and posterior vaginal walls approximate. If you are unsure, it is best not to label it.

The left-hand example actually shows collapse of the anterior and posterior lower segment myometrium against each other and not true cervix despite the label by the sonologist. Nevertheless, we would want this to be labelled as cervix for annotation purposes.

Note the hyperechogenic line in the right-hand image that is from the anterior and posterior vaginal walls approximating.

## Steps 2-3

- Do not label frames where the image has blurred as the probe was put on or lifted off.
- Bounding boxes need to be drawn around the cervix, vagina, bladder and placenta in step 3.1 and 3.2, following the same principles as above.
- Instead of bounding boxes, marker boxes can be drawn down the right-hand side of the window for all other structures although if convinced about views of the cerebellum and stomach bubble, bounding boxes around these would be helpful.

*All images are taken by the authors.*
